# Exploring the potential of blood and urine protein electrophoresis in predicting 1-year mortality in acute kidney injury

**DOI:** 10.1371/journal.pone.0327044

**Published:** 2025-06-25

**Authors:** Deniz Yilmaz, Ezgi Sahin, Gizem Batar, Koray Caglayan, Emine Gulturk, Sengul Aydin Yoldemir

**Affiliations:** 1 Department of Internal Medicine, Bakirkoy Dr. Sadi Konuk Training and Research Hospital, Istanbul, Turkiye; 2 Department of Rheumatology Clinic, Istanbul University Faculty of Medicine, Istanbul, Turkiye; 3 Department of Hematology, Bakirkoy Dr. Sadi Konuk Training and Research Hospital, Istanbul, Turkiye; 4 Department of Internal Medicine, Kanuni Sultan Suleyman Training and Research Hospital, Istanbul, Turkiye; Versiti Blood Research Institute, UNITED STATES OF AMERICA

## Abstract

This retrospective cohort study aimed to assess the predictive value of protein fractions obtained from blood and urine protein electrophoresis, along with various clinical and laboratory parameters, for 1-year all-cause mortality in acute kidney injury (AKI) patients. Data were collected from hospitalized patients who had been diagnosed with AKI. Demographics, smoking status, blood and urine electrophoresis results, serum gamma globulin levels, monoclonal gammopathy status, immunofixation results, serum free kappa (κ) and lambda (λ), and urine κ and λ levels were measured in addition to routine biochemistry and complete blood counts. In addition, serum free κ-to-λ ratio and urine κ-to-λ ratio were calculated. The primary endpoint was 1-year all-cause mortality and its association with electrophoresis-obtained data. Among the 295 patients included in the analyses, 65 (22.03%) experienced mortality, with higher mean age (72.75 ± 13.51) compared to the survival group (62.58 ± 16.59) (p < 0.001). Sex distribution showed no significant difference between groups. No significant disparities were observed in electrophoresis parameters and other laboratory values. Multivariable logistic regression showed that high age (OR: 1.038, 95% CI: 1.016–1.062, p = 0.001) and low albumin (OR: 0.450, 95% CI: 0.263–0.770, p = 0.004) were independent predictors of mortality. We find that the evidence gathered in the present study is insufficient to recommend the use of blood and urine protein fractions for diagnostic or prognostic purposes in patients with AKI. Nonetheless, the current data showing some notable variations in urine κ and λ levels suggest that further studies are warranted to explore this relationship.

## Introduction

Acute kidney injury (AKI) is characterized by sudden but usually reversible decrease in glomerular filtration rate (GFR) [1]. Despite reversibility in the majority of cases, AKI is common among hospitalized patients (14%) and is associated with heightened risks for mortality [2].

Since the triggering factor for AKI can be difficult to pinpoint, it may be useful to utilize severity-related markers or risk factors to guide management [2]. These risk factors associated with mortality can allow for stratification of high-risk patients [3,4]. Such prognostic factors have been shown to yield reliable results in AKI assessment, especially in the acute period [3,5–7]; however, there are very few accurate prognostic factors applicable for the long-term risk stratification of patients with AKI [6,8].

The free light chains (FLCs) of immunoglobulin structures (that are normally disposed of by the kidneys) accumulate in the circulation as a result of reduced renal function [9–11]. In fact, literature demonstrated that elevated FLC level can be indicative of poor renal function, advanced stages of various diseases, and increased risk of mortality (due to renal function or otherwise) [12–14]. Although a number of ratios calculated from urine or serum protein levels may be prognostic in patients with acute renal diseases [15], data is limited in terms of relationships with AKI outcomes [15]. It must be emphasized that protein electrophoresis results are also altered by monoclonal gammopathies or plasma cell disorders, but these could also reflect kidney myeloma, which may be a presentation of AKI [16,17]. While monoclonal gammopathies are a relatively rare cause of AKI, they are associated with systemic inflammation, endothelial dysfunction, and altered immune responses, which may contribute to worse outcomes in these patients. Furthermore, individuals with high levels of monoclonal gammopathy reportedly experience worse prognosis than others [16–18]. Taken together, it appears that altered levels of protein fractions and the resultant ratios could provide valuable prognostic information for diagnostic and prognostic purposes in AKI. Owing to limited research in this specific topic, the exact role of protein electrophoresis results in predicting medium- and long-term mortality in patients with AKI remains unclear.

The main purpose of this study was to investigate whether the concentrations of protein fractions obtained in blood and urine protein electrophoresis can predict 1-year all-cause mortality in patients with AKI. As a secondary aim, we investigated whether other clinical and laboratory parameters could predict 1-year all-cause mortality.

## Materials and methods

### Study design, participants and data collection

This study included patients who were hospitalized with AKI diagnosis at the İnternal Medicine Department of Bakirkoy Dr Sadi Konuk Hospital from January 2020 to December 2023. The data for this study were accessed for research purposes during the period from 17 April 2023 to 1 December 2023. The analyses conformed to a retrospective cohort design. Individuals below the age of 18, individuals diagnosed with or having a history of malignancy, participants with a follow-up duration of less than one year and individuals whose death had definitively occurred due to a distinct and identifiable cause (such as trauma) were excluded. Additionally, postrenal causes were carefully evaluated and excluded during the clinical assessment and individuals were excluded if they had missing core demographic data (age, sex), mortality status, or basic laboratory parameters required for primary analysis. All researchers in the study followed the principles of the Declaration of Helsinki and the study was begun after receiving approval from the Clinical Ethics Committee of Bakirkoy Dr. Sadi Konuk Training and Research Hospital with respect to the same principles (Decision date:17.04.2023, decision no: 2023-08-08).

Patients’ data, including age, sex and comorbidity information, smoking status, blood and urine electrophoresis results, and other laboratory measurements at the time of AKI diagnosis were recorded. In addition, patient files were reviewed to collect other data, such as length of hospital stay and mortality status.

### Endpoints and definitions

The primary endpoint of the study was all-cause mortality during 1 year of follow-up subsequent to initial AKI detection. The diagnosis and management of AKI was based upon the guidelines outlined in the 2012 Kidney Disease: Improving Global Outcomes (KDIGO) document [19], specifically with respect to the established creatinine criteria [19]. The baseline estimated GFR was determined through the application of the Modification of Diet in Renal Disease equation, incorporating appropriate adjustments tailored for female patients [20].

### Laboratory analyses

All laboratory measurements were made in the Biochemistry Department of our hospital according to the manufacturer’s instructions using calibrated standard measuring devices. Patients’ creatinine, albumin, calcium, C-reactive protein (CRP), erythrocyte sedimentation rate, ferritin, and complete blood count values (including hemoglobin, hematocrit, mean corpuscular volume levels and absolute white blood cell, lymphocyte, neutrophil and platelet counts) were collected. Serum creatinine levels were analyzed by the modified Jaffe method in a Cobas 8000 analyzer (Roche, Mannheim, Germany). As part of standard practice, serum protein electrophoresis is routinely performed for patients admitted with AKI, and follow-up tests (FLC, immunofixation) are automatically triggered by abnormal results.

Gamma globulin levels and the presence of monoclonal peak were determined by capillary zone electrophoresis. Immunofixation was performed on Automated System for electrophoresis (Via Rina Monti, Roma, Italy). Hypogammaglobulinemia was defined as an immunoglobulin G level significantly lower than the lower threshold of the reference range (7–16 g/L) [21]. Monoclonal gammopathy status (absent or present) and immunofixation band status (absent or present) were recorded.

FLC κ and λ levels in serum and 24-hour urine were measured by the immunoturbidimetric method (The Binding Site Group Ltd, Birmingham, UK) using the BNII nephelometer (Siemens Diagnostics; Marburg, Germany). FLC κ-to-FLC λ ratio was calculated separately for serum and urine using individual patient values rather than dividing the mean of one group by the mean of the other. The manufacturer-provided reference ranges for serum free light chains were as follows: FLC κ: 3.3–19.4 mg/L, FLC λ: 5.71–26.3 mg/L, and FLC κ-to-FLC λ ratio: 0.26–1.65. For urinary free light chains, the reference values were <32.9 mg/L for FLC κ and <3.79 mg/L for FLC λ. While established reference ranges for serum FLC levels exist, standardized reference values for urinary FLC levels and their ratios are not well defined [22]. Serum and urine free light chain measurements were performed as part of routine clinical practice when protein electrophoresis results were abnormal, rather than systematically for all patients. These measurements were available for 134 of 295 patients (45.4%) for serum free light chains and 43 of 295 patients (14.6%) for urine free light chains.

### Statistical analysis

Statistical significance was set at a p value of 0.05. All analyses were performed on SPSS for Windows, v25.0 (IBM, Armonk, NY, USA). Histograms and Q-Q plots were used to examine the conformity of the variables to normal distribution. Descriptive statistics for categorical data were presented with relative and absolute frequencies, and comparisons were performed with relevant Chi-square tests (Pearson, Yate’s correction, Fisher’s exact). Numerical data was described by mean ± standard deviation for normally distributed variables, and these were compared via the independent samples t-test. Descriptives for non-normally distributed continuous variables were median (25th percentile – 75th percentile), and these were analyzed with the Mann-Whitney U test. Logistic regression was performed to identify factors independently associated with mortality. The multivariable model included variables that demonstrated significance in univariable regression.

Missing data patterns were assessed for free light chain measurements. All analyses clearly specify the number of patients with available data for each parameter. Primary analyses were conducted on the complete dataset (n = 295) for protein electrophoresis parameters, while free light chain analyses were performed on available subsets and treated as exploratory.

## Results

**Fig 1:**
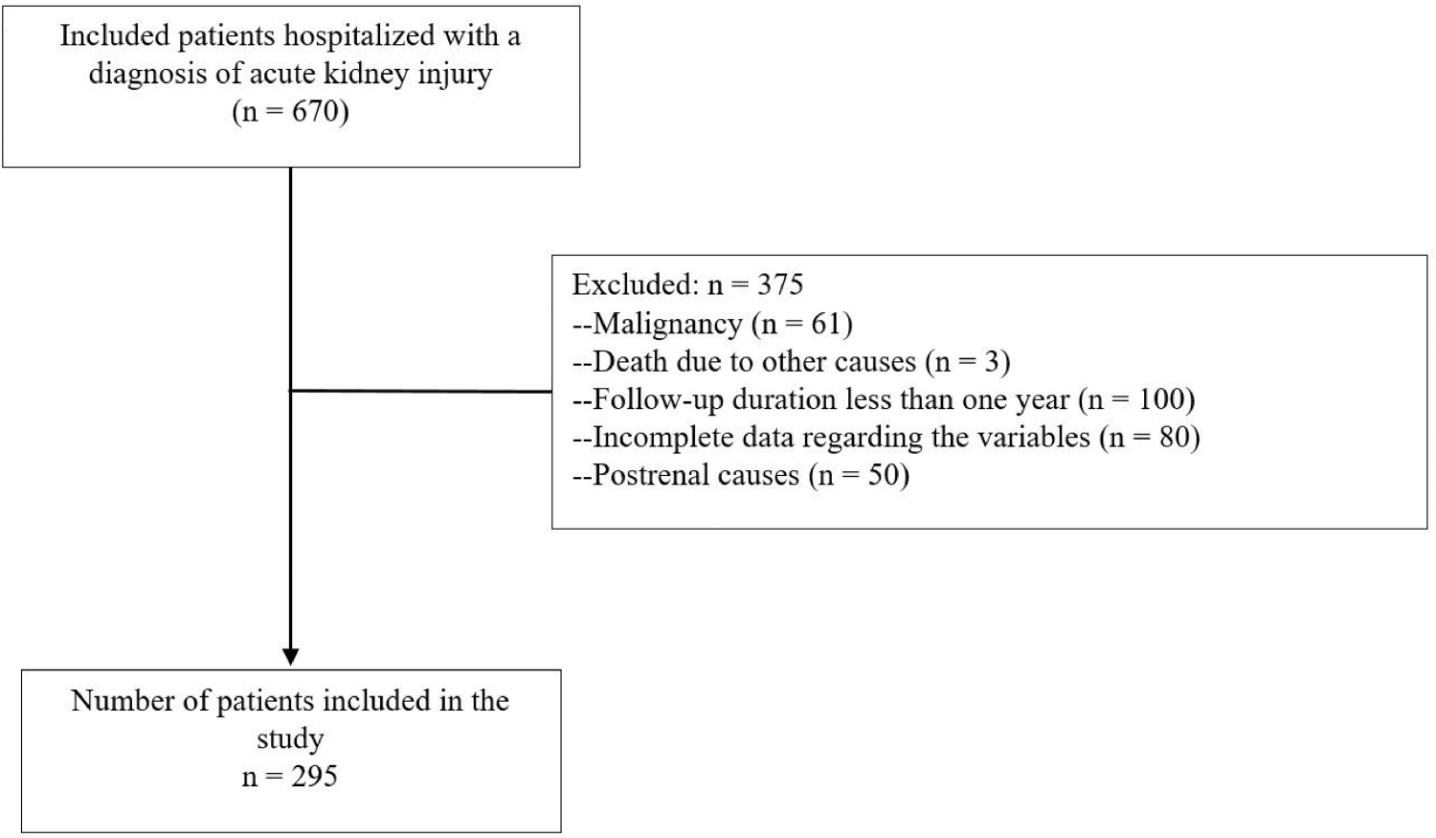
Flowchart of the study.

A total of 670 patients hospitalized with a diagnosis of AKI in the nephrology clinic during the study dates were evaluated for eligibility. Among these 375 were excluded due to meeting exclusion criteria. The remaining 295 patients were included in the analyses (Fig 1).

Sixty-five (22.03%) of the enrolled patients were categorized in the mortality group, whereas 230 individuals (77.97%) were classified in the survival group. The mean age of the mortality group (72.75 ± 13.51) was observed to be significantly higher than that of the survival group (62.58 ± 16.59) (p < 0.001). Sex distribution revealed that 52.31% of the mortality group and 56.96% of the survival group were male, but this disparity was not significant (p = 0.505). The prevalence of congestive heart failure was markedly higher in the mortality group (p = 0.001). Furthermore, the duration of hospital stay was significantly longer among survivors compared to the mortality group. No other significant differences were identified between the two groups with respect to clinical variables, electrophoresis parameters, or other laboratory metrics. Free light chain data were available for a subset of patients based on clinical indication (abnormal protein electrophoresis results). No significant differences in baseline characteristics or mortality were observed between patients with and without available free light chain measurements. (Table 1).

**Table 1:** Demographics, laboratory measurements and electrophoresis results with regard to mortality.

	Total (n = 295)	Mortality		
No (n = 230)	Yes (n = 65)	p
Age	64.82 ± 16.49	62.58 ± 16.59	72.75 ± 13.51	**<0.001**
Sex				
Male	165 (55.93%)	131 (56.96%)	34 (52.31%)	0.505
Female	130 (44.07%)	99 (43.04%)	31 (47.69%)	
Comorbidities				
Previous chronic renal failure	119 (40.34%)	87 (37.83%)	32 (49.23%)	0.098
Diabetes mellitus	148 (50.17%)	118 (51.30%)	30 (46.15%)	0.463
Hypertension	221 (74.92%)	166 (72.17%)	55 (84.62%)	0.060
Coronary artery disease	87 (29.49%)	62 (26.96%)	25 (38.46%)	0.072
Congestive heart failure	56 (18.98%)	36 (15.65%)	20 (30.77%)	**0.010**
Cerebrovascular disease	25 (8.47%)	19 (8.26%)	6 (9.23%)	1.000
COPD	27 (9.15%)	20 (8.70%)	7 (10.77%)	0.788
Thyroid diseases	12 (4.07%)	8 (3.48%)	4 (6.15%)	0.306
Rheumatic diseases	10 (3.39%)	9 (3.91%)	1 (1.54%)	0.697
Smoking	66 (22.37%)	51 (22.17%)	15 (23.08%)	1.000
Creatinine, mg/dL	2.6 (1.6 - 4.2)	2.59 (1.6 - 4.5)	3.0 (1.9 - 3.8)	0.757
Albumin, g/dL	3.29 ± 0.59	3.35 ± 0.57	3.07 ± 0.61	**0.001**
Calcium, mg/dL	8.30 ± 0.96	8.31 ± 0.97	8.24 ± 0.92	0.602
CRP, mg/L	35 (11 - 89)	33 (11 - 87)	43 (16 - 105)	0.205
Erythrocyte sedimentation rate, mm/h	42 (22 - 69)	42 (23 - 68)	36 (17 - 70)	0.418
Ferritin, ng/mL	228 (126 - 510)	231 (127 - 496)	203 (126 - 564)	0.605
Hemoglobin, g/dL	9.58 ± 1.93	9.69 ± 1.97	9.19 ± 1.77	0.065
Hematocrit, %	29.52 ± 5.50	29.84 ± 5.59	28.40 ± 5.06	0.062
MCV, fL	86.03 ± 8.15	85.94 ± 7.92	86.37 ± 8.97	0.707
WBC (x103)	8.61 ± 3.97	8.60 ± 4.05	8.66 ± 3.71	0.914
Lymphocyte (x103)	1.39 ± 0.67	1.43 ± 0.68	1.25 ± 0.60	0.051
Neutrophil (x103)	6.44 ± 3.62	6.42 ± 3.73	6.51 ± 3.21	0.855
Platelet (x103)	249.79 ± 112.32	254.03 ± 116.05	234.53 ± 97.04	0.220
Gamma globulin	10.52 ± 3.97	10.39 ± 3.83	11.00 ± 4.44	0.277
Hypogammaglobulinemia	82 (27.80%)	65 (28.26%)	17 (26.15%)	0.673
Normal	152 (51.53%)	120 (52.17%)	32 (49.23%)	
Hypergammaglobulinemia	61 (20.68%)	45 (19.57%)	16 (24.62%)	
Monoclonal gammopathy				
Absent	264 (89.49%)	208 (90.43%)	56 (86.15%)	0.444
Present	31 (10.51%)	22 (9.57%)	9 (13.85%)	
Immunofixation				
Band absent	132 (80.49%)	102 (81.60%)	30 (76.92%)	0.680
Band present	32 (19.51%)	23 (18.40%)	9 (23.08%)	
Serum free kappa, mg/L	97.54 ± 50.97	98.96 ± 53.71	93.86 ± 43.54	0.608
Serum free lambda, mg/L	122.12 ± 59.93	121.78 ± 58.18	123.03 ± 65.17	0.916
Serum free kappa-to-lambda ratio	0.79 (0.66 - 0.93)	0.79 (0.67 - 0.93)	0.79 (0.65 - 0.93)	0.839
Urine kappa, mg/L	65.0 (31.5 - 103.0)	61.8 (29.7 - 103.0)	80.55 (57.0 - 103.5)	0.218
Urine lambda, mg/L	30.4 (17.0 - 58.0)	28.9 (9.26 - 60.5)	41.1 (22.55 - 56.15)	0.244
Urine kappa-to-lambda ratio	2.13 (1.76 - 2.43)	2.20 (1.73 - 2.54)	1.95 (1.81 - 2.27)	0.597
Length of stay in hospital, days	13 (10 - 15)	13 (11 - 14)	9 (5 - 15)	**<0.001**

Descriptive statistics were presented by using mean ± standard deviation for normally distributed continuous variables, median (25th percentile – 75th percentile) for non-normally distributed continuous variables, and frequency (percentage) for categorical variables.

Free light chain measurements: Serum κ and λ available for 134 patients (45.4%); urine κ and λ available for 43 patients (14.6%)

Abbreviations; COPD: Chronic obstructive pulmonary disease, CRP: C-reactive protein, MCV: Mean corpuscular volume, WBC: White blood cell

Univariate logistic regression analysis yielded significant associations between mortality and age [Odds Ratio (OR) (95% Confidence Interval (CI)) = 1.047 (1.025–1.070), p < 0.001], hypertension [OR (95% CI) = 2.120 (1.019–4.413), p = 0.044], congestive heart failure [OR (95% CI) = 2.395 (1.269–4.522), p = 0.007], and albumin level [OR (95% CI) = 0.427 (0.259–0.704), p = 0.001] (Table 2).

**Table 2:** Odds ratios for mortality, logistic regression analysis.

	Univariable		Multivariable	
	OR (95% CI)	p	OR (95% CI)	p
Age	1.047 (1.025 - 1.070)	**<0.001**	1.038 (1.016 - 1.062)	**0.001**
Sex, Female	1.206 (0.694 - 2.096)	0.505		
Previous chronic renal failure	1.594 (0.915 - 2.775)	0.099		
Diabetes mellitus	0.814 (0.468 - 1.413)	0.464		
Hypertension	2.120 (1.019 - 4.413)	**0.044**	1.309 (0.591 - 2.902)	0.507
Coronary artery disease	1.694 (0.950 - 3.020)	0.074		
Congestive heart failure	2.395 (1.269 - 4.522)	**0.007**	1.826 (0.930 - 3.587)	0.080
Cerebrovascular disease	1.129 (0.431 - 2.956)	0.804		
COPD	1.267 (0.511 - 3.144)	0.609		
Thyroid diseases	1.820 (0.530 - 6.246)	0.341		
Rheumatic diseases	0.384 (0.048 - 3.085)	0.368		
Smoking	1.053 (0.547 - 2.028)	0.877		
Creatinine	0.949 (0.831 - 1.083)	0.437		
Albumin	0.427 (0.259 - 0.704)	**0.001**	0.450 (0.263 - 0.770)	**0.004**
Calcium	0.926 (0.694 - 1.236)	0.601		
CRP	1.001 (0.998 - 1.005)	0.470		
Erythrocyte sedimentation rate	0.998 (0.989 - 1.007)	0.645		
Ferritin	1.000 (1.000 - 1.001)	0.661		
Hemoglobin	0.869 (0.748 - 1.010)	0.067		
Hematocrit	0.952 (0.903 - 1.003)	0.063		
MCV	1.007 (0.973 - 1.042)	0.705		
WBC (x10^3^)	1.004 (0.937 - 1.076)	0.914		
Lymphocyte (x10^3^)	0.637 (0.405 - 1.004)	0.052		
Neutrophil (x10^3^)	1.007 (0.934 - 1.086)	0.855		
Platelet (x10^3^)	0.998 (0.996 - 1.001)	0.220		
Gamma globulin	1.038 (0.971 - 1.110)	0.277		
Gamma globulin level^(1)^				
Hypogammaglobulinemia	0.981 (0.506 - 1.900)	0.954		
Hypergammaglobulinemia	1.333 (0.668 - 2.661)	0.415		
Monoclonal gammopathy, Present	1.519 (0.663 - 3.484)	0.323		
Immunofixation, Band present	1.330 (0.557 - 3.180)	0.521		
Serum free kappa	0.998 (0.990 - 1.006)	0.605		
Serum free lambda	1.000 (0.994 - 1.007)	0.915		
Serum free kappa-to-lambda ratio	1.167 (0.353 - 3.862)	0.800		
Urine kappa	1.003 (0.992 - 1.015)	0.570		
Urine lambda	1.003 (0.984 - 1.022)	0.755		
Urine kappa-to-lambda ratio	0.688 (0.276 - 1.712)	0.421		
Nagelkerke R^2^	–		0.166	

(1) Reference category: Normal

Abbreviations; CI: Confidence interval, COPD: Chronic obstructive pulmonary disease, CRP: C-reactive protein, MCV: Mean corpuscular volume, OR: Odds ratio, WBC: White blood cell

Multivariable logistic regression analysis, adjusting for various covariates, reaffirmed that advanced age (OR: 1.038, 95% CI: 1.016–1.062, p = 0.001) and reduced albumin levels (OR: 0.450, 95% CI: 0.263–0.770, p = 0.004) were independently associated with increased mortality risk (Table 2).

## Discussion

Although several established biomarkers have demonstrated some predictive value for AKI outcomes, their accuracy remains suboptimal, and there is still a need for additional prognostic tools. Plasma protein electrophoresis was explored in this study due to its ability to provide a broad overview of protein distribution, which has been suggested to reflect underlying inflammatory and renal pathophysiological processes. Given prior reports linking protein fractions to kidney function [1,22–25]., we aimed to assess whether specific alterations in plasma protein patterns could serve as prognostic markers in AKI. However, our findings indicate that in this study, plasma electrophoresis parameters did not demonstrate a predictive association with 1-year mortality. Notably, older age and low albumin were the only two factors clarified as independent risk factors.

### Plasma proteins and mortality in AKI

Despite progress in diagnosing and treating AKI, the mortality associated with it remains elevated. Consequently, the KDIGO guidelines recommend the monitoring of all AKI patients [19] and the UK guidelines recommend 2–3 years of post-AKI surveillance [4]. In addition to the traditional mortality risk factors in AKI, which will be discussed below, systemic inflammation is also thought to play an important role in the pathogenesis and prognosis of AKI [15,26–28]. Given that immunoglobulin FLC levels are potential markers of immune system activation [15], it has been hypothesized that alterations in protein fractions might be linked to AKI prognosis. However, our findings did not support this hypothesis. This discrepancy may be attributed to several factors, including the limited subset of patients with available FLC data (45.4% for serum, 14.6% for urine), potential selection bias due to clinical indication-based testing, and insufficient statistical power to detect modest associations.

Prior studies suggested a possible association between serum protein distribution and renal disease severity [16], but their applicability to general AKI populations remains questionable. For instance, a study by Wang et al. reported that elevated combined FLC concentrations independently predicted mortality in AKI patients post-cardiovascular surgery [15]. However, our study did not confirm such an association in a broader AKI cohort. Some other previous studies have also shown that serum FLC concentration is associated with increasing stage in patients with chronic renal failure [9–11]. Nonetheless, the absence of large-scale longitudinal studies with homogeneous patient populations and long follow-up periods makes it difficult to draw definitive conclusions.

### Urine protein findings

AKI commonly stems from damage to renal structures, triggered by factors such as sepsis, hypotension, drug toxicity, and cardiopulmonary events [29]. Although decreased urine output and increased serum creatinine are the most important distinguishing features of AKI, there is insufficient evidence regarding the levels of proteins in urine and their direct relationship with AKI severity. Previous research has indicated that urinary protein patterns might have prognostic value in AKI, with some studies suggesting their potential role in predicting renal failure progression [2,30]. However, despite greater variations in urine values compared to serum, our findings did not support an association between urine FLC κ and λ levels or the FLC κ-to-λ ratio with mortality after AKI. In a general sense, the literature supports the assertion that urine protein patterns can be prognostic in patients with AKI. However, despite relatively greater variations in urine values (compared to serum values), we found that neither urine FLC κ and λ nor FLC κ-to-λ ratio were associated with mortality after AKI. Although urine protein patterns have the potential for AKI prognostication, AKI is a disease that has unforecastable characteristics and numerous etiologies. These biases could impact the alteration of urinary proteins, especially with respect to the longitudinal properties of kidney injury.

The variability in the properties and etiologies of AKI make it difficult to create homogeneous patient groups and determine generalizable prognostic factors [1]. Research has sought to identify patient risk stratification markers in order to be able to generate strategies for treatment early in the course of the disease [3,5–7]. However, prognosis predictors for the medium and long term are rather weak. In our study, advanced age and low albumin levels were found to be independent risk factors for 1-year mortality. Risk factors reported in previous publications on mortality after AKI include previous AKI history, AKI severity, baseline renal function levels, and subsequent recovery of renal function [3,5–7]. In a large population-based cohort study, in patients who developed AKI in the hospital, the factors most associated with mortality in the mid- and long-term after discharge were reported to be initial renal function, AKI severity, and previous AKI history [3]. In the study of Wang et al., age, aortic surgery, high creatinine, high CRP, and decreased albumin were determined to be risk factors associated with 90-day mortality among subjects suffering from AKI following cardiac operations [15]. Also there are a number of parameters worthy of mention in this respect, including cystatin C [31], neutrophil gelatinase associated lipocalin [32] and urinary liver-type fatty acid-binding protein [33].

### Clinical implications

In general, AKI poses a significant clinical challenge with a notable mortality rate, and effective prognostic markers for medium to long-term mortality are currently lacking. While some studies have shown promising results regarding the predictive value of blood and urine protein patterns for AKI prognosis, our study did not yield significant findings in this context. Notably, advanced age and low albumin levels emerged as potential prognostic markers for AKI. We must also record the fact that urine levels of FLC markers suggested considerable variations in some patients; however, prospectively desgined studies that can leverage the heteregenous inclusion of patients in order to characterized potential subgroups are necessary to clarify whether there exist distinct subgroups in which these markers can be utilized. This, in turn, may aid in differentiating high-risk patients in terms of post-AKI mortality, ultimately contributing to the prevention of mortality associated with the condition.

The study under consideration presents several limitations that warrant careful interpretation of its findings. The foremost limitation lies in its retrospective design and the restriction to a single center, which may limit the generalizability of the results to a broader population. Beyond these primary constraints, additional possible limitations should be acknowledged, which may influence the comprehensive understanding of the study’s outcomes.

### Limitations

One notable limitation of our study is the heterogeneous etiology of AKI. While stratifying patients based on the underlying cause of AKI would have been ideal, this was not feasible due to the retrospective design and the available dataset, which lacked comprehensive documentation on AKI etiologies. The inability to differentiate between ischemic, toxic, septic, or other causes of AKI limits our ability to determine whether specific prognostic markers hold significance within particular subgroups. Additionally, although we reported comorbidities, we did not analyze specific causes of death. Given that AKI-related mortality is multifactorial, evaluating all-cause mortality without distinguishing deaths directly attributable to AKI may have diluted potential associations between protein electrophoresis parameters and outcomes. For instance, patients with severe AKI but who ultimately died from unrelated conditions, such as cardiovascular events, could obscure true relationships within an AKI-specific cohort. Another limitation is the absence of time-to-event data, as the exact duration between AKI onset and mortality was unavailable for many patients. This prevented us from conducting survival analyses, which could have provided insight into whether certain biomarkers are more relevant for early versus late mortality. Moreover, our reliance on hospital records for mortality data introduces potential underreporting, as some post-discharge deaths may not have been captured. Although these records are generally well-maintained, the lack of external validation (e.g., national death registries) means that some events could have been missed, particularly in patients who were lost to follow-up. A key aspect of our study design was to assess the prognostic value of baseline plasma and urinary protein fractions in AKI patients. However, we acknowledge that serial measurements over time could provide additional insights into dynamic changes and their potential impact on prognosis. Given that AKI is a heterogeneous and evolving condition, tracking protein fraction levels longitudinally might help identify trends associated with recovery or progression. Not all patients had available serum/urine free light chain data, representing an important limitation. The availability of serum and urine free light chain data was limited to a subset of patients rather than the entire study population. While these measurements were included as exploratory analyses, their incomplete nature may affect the generalizability of results related to free light chain parameters. However, it’s important to note that all primary analyses were based on complete datasets, and the free light chain measurements were obtained systematically without regard to clinical outcomes. The study did not evaluate other serum protein electrophoresis fractions (e.g., alpha, beta), which may have additional prognostic value in AKI mortality prediction. Future studies incorporating serial measurements could further clarify the temporal relationship between these biomarkers and clinical outcomes, potentially refining their prognostic utility. These limitations should be considered when interpreting the findings and highlight the need for future prospective studies with more granular data collection.

## Conclusion

Our data shows that blood and urine protein electrophoresis results measured at AKI diagnosis did not significantly predict 1-year all-cause mortality among patients with AKI. Independent risk factors for mortality were older age and low albumin levels. Urine FLC data yielded a greater level of variation compared to serum data when compared between patients with and without mortality. Although the study provides valuable insights and a comprehensive dataset, the lack of a direct relationship concerning the electrophoresis data and mentioned limitations must be cautiously assessed when drawing interpretations. Future research in this field should consider addressing these limitations to enhance the depth and breadth of our understanding of prognostic markers and mortality factors in patients with AKI.
